# Vestibular Function in Long-Term Hearing Aid Users: A Preliminary Investigation

**DOI:** 10.3390/audiolres16010010

**Published:** 2026-01-15

**Authors:** M. Ramiz Malik, Kaushlendra Kumar, Mohan Kumar Kalaiah, Niraj Kumar Singh, Mayur Bhat

**Affiliations:** 1 Department of Audiology and Speech Language Pathology, Kasturba Medical College Mangalore, Manipal Academy of Higher Education, Manipal 576104, India; ramizmalik.m@gmail.com (M.R.M.); kaushlendra.kumar@manipal.edu (K.K.);; 2 Department of Audiology, All India Institute of Speech and Hearing, Manasagangothri, Mysuru 570006, India

**Keywords:** vestibular function, otolithic function, vestibular evoked myogenic potential, hearing aids, sensorineural hearing loss

## Abstract

**Background/Objectives:** This study compared vestibular evoked myogenic potentials (VEMP) and video head impulse test (vHIT) findings between long-term hearing aid users and non-users with moderately severe to profound sensorineural hearing loss (SNHL) to investigate whether prolonged use of high-power hearing aids has any effect on the functioning of peripheral vestibular organs. **Methods:** A cross-sectional study was conducted in the audiology clinic of a tertiary care hospital. Using convenience sampling, 67 adults aged 20–64 years who visited for hearing evaluation or hearing aid services were recruited and allocated into hearing aid user and non-user groups. VEMP latency and amplitude and vHIT vestibulo-ocular reflex (VOR) gain values were compared between groups. Multiple linear regression was performed to examine the association between the duration of hearing aid use and vestibular function. **Results:** No significant group differences were observed for any VEMP or vHIT parameter, except for the latency of P1 of the cVEMP in the left ear. Regression analyses indicated that the duration of hearing aid use was not significantly associated with any vestibular test measure. **Conclusions:** Long-term use of high-power hearing aids does not appear to be associated with measurable alterations in vestibular function. Nonetheless, longitudinal studies with improved control of confounding variables are recommended to validate these preliminary findings.

## 1. Introduction

The inner ear consists of the cochlea and the peripheral vestibular end organs. The cochlea is the organ of hearing, while the vestibular apparatus is responsible for gaze stabilization and postural control by detecting gravity. Both the cochlea and vestibular apparatus are similar in many aspects. Embryologically, both organs developed from the same structure, the otic placode of the ectoderm [1,2]. There is also similarity in the ultrastructure of sensory receptor cells and their functioning in both systems. In addition, they are innervated by branches of the eighth cranial nerve (the vestibulo-cochlear nerve) and blood supplied by the same labyrinthine artery, a branch of the anterior inferior cerebellar artery [1,2,3,4].

Given these close anatomical and physiological connections, it is plausible that factors affecting cochlear function may similarly influence vestibular function [4,5]. Conditions such as endolymphatic hydrops or labyrinthine infections always produce both auditory and vestibular symptoms. Sustained acoustic overstimulation has also been recognized as a potential factor impacting both systems. Evidence of vestibular damage in individuals with chronic noise exposure and the presence of vestibular evoked myogenic potentials (VEMPs) in response to high-intensity acoustic stimuli supports the notion that sufficiently intense sounds can stimulate the vestibular end organs as well. Animal studies have reported cellular-level damage to vestibular organs after noise exposure, and the pattern of damage is similar to that observed in the cochlea [6,7]. The hair cells of the vestibular organs are morphologically similar to cochlear hair cells and are susceptible to mechanical and metabolic stress. Prolonged high-intensity sound stimulation can generate excessive fluid motion within the vestibule, leading to disruption of the otolithic membrane and cellular damage due to oxidative stress and excitotoxicity [6,7]. These mechanisms provide a physiological basis for the observed vestibular effects of acoustic overstimulation. In humans, the vestibular assessment used to study the impact of acoustic overstimulation typically uses tools like VEMP and caloric tests [8].

Hearing aids are sound amplification devices designed to improve audibility of sounds for individuals with hearing loss. Fitting hearing aids is a vital part of aural rehabilitation, as it enhances speech perception, communication skills, and the quality of life of individuals with hearing loss [9]. Despite their benefits, concerns exist regarding the potential adverse effects of hearing aids on residual hearing due to high output levels, but research findings are inconclusive [10,11,12]. Modern hearing aids can produce output levels up to 132 dB SPL, which exceeds the recommended safe listening limits [13,14]. Some studies suggest that long-term hearing aid use may negatively impact cochlear function, evidenced by reduced otoacoustic emissions and elevated hearing thresholds [15,16].

As discussed earlier, the cochlea is not the only organ at risk for damage due to acoustic overstimulation; similar effects on vestibular function in hearing aid users are plausible too. However, the literature reports show improved balance with hearing aid use rather than deterioration [17,18]. These findings were justified by the assumption of better spatial orientation with a hearing aid from improved hearing than the condition of no hearing. While this may be true, these measures were reported either immediately or within only a few days of the hearing device fitting. Nonetheless, acoustic overstimulation requires a prolonged period to show its detrimental impact, which was not assessed in these studies. It remains to be investigated whether prolonged hearing aid use leads to measurable vestibular alterations, especially in the otolithic organs (saccule and utricle), which are anatomically closer to the cochlea and are more prone to damage from acoustic overstimulation [8].

Importantly, to the best of our knowledge, no previous study has systematically compared vestibular function between long-term hearing aid users and non-users with sensorineural hearing loss. Thus, the present study represents the first objective evaluation of both otolithic and semicircular canal function in long-term hearing aid users, with direct comparison to non-users.

Given the established utility of VEMP and video head impulse test (vHIT) in evaluating the effects of sustained sound exposure [19,20,21], the present study aimed to assess vestibular function in long-term hearing aid users using these objective measures. The specific objectives were to compare otolithic function between long-term hearing aid users and non-users with sensorineural hearing loss using cervical and ocular vestibular evoked myogenic potentials (cVEMP and oVEMP) and to compare semicircular canal function between the two groups using the vHIT. Additionally, the study examined the association between the duration of hearing aid use and vestibular test parameters.

## 2. Materials and Methods

The present cross-sectional comparative study was carried out in the Department of Audiology and Speech Language Pathology at a tertiary care hospital in Mangalore, India, after obtaining approval from the Institutional Ethics Committee (IEC No. IECKMCMLR-12/2022/493).

### 2.1. Participants

The participants were selected from adults with hearing loss who visited the hospital for audiology services such as hearing assessment, hearing aid fitting, or reprogramming. Among those willing to participate, a pre-selection assessment was conducted following the department’s standard operating protocol. This included detailed audiological case history, otoscopic examination, immittance evaluation, and pure-tone audiometry.

Individuals with moderately severe to profound sensorineural hearing loss were then provided with a patient information sheet, and written informed consent was obtained. Subsequently, a basic vestibular evaluation was conducted, which included detailed vestibular case history and a series of subjective vestibular tests. The subjective tests administered were the Tandem Gait Test, Fukuda Stepping Test, Romberg’s Test, and head impulse test. In addition, oculomotor function evaluation was carried out using videonystagmography (VNG).

Adults aged between 20 and 64 years with acquired moderately severe to profound sensorineural hearing loss were recruited for the study. Individuals with a history of middle-ear pathology, neurological disorders, or obvious vestibular pathologies (such as Ménière’s disease, benign paroxysmal positional vertigo, vestibular neuritis, or central vestibular dysfunction), as well as those with a history of vestibulotoxic medication use or prior exposure to hazardous noise, were excluded. Given these criteria and the post-lingual nature of hearing loss in the participants, the most plausible etiologies for the present sample are early or typical presbycusis and idiopathic sensorineural hearing loss.

Participants were then categorized into two groups based on hearing aid usage. The first group (hearing aid users) consisted of individuals who had been fitted with digital behind-the-ear (BTE) or receiver-in-the-canal (RIC) hearing aids with high-power receivers for at least one year, with gain appropriately set according to their degree of hearing loss. They were regular users who wore their hearing aids for a minimum of 8 hours per day. The second group (non-users) included individuals who had never used hearing aids prior to their enrolment in the study. Participants in both groups were matched for age, gender, degree of hearing loss, and duration of hearing loss.

### 2.2. Hearing Aid Real Ear Measurement and Exposure Estimation

Quantifying the potential acoustic exposure delivered to the ear canal through hearing aid use was an essential component of this study. Because real world hearing aid output varies dynamically with changes in input level, acoustic environment, background noise, and device processing, it was not feasible in the present study to capture true everyday exposure levels. Therefore, a standardized laboratory based real ear measurement procedure was adopted to obtain a controlled and comparable estimate of possible exposure. This procedure was adapted from methods used in studies on personal listening devices, with modifications appropriate for hearing aid evaluation [19,20,21].

Hearing aid real ear measurements were obtained using Audioscan verifit real ear measurement system (Etymonic Design Inc., Dorchester, ON, Canada) in a standard audiometric room with ambient noise maintained within acceptable clinical limits. Each participant’s hearing aid had been previously programmed and optimized during routine clinical fitting, and all measurements for the current study were conducted using the same program and volume settings that the participant used in daily life. The probe tube was inserted approximately 28 mm into the ear canal (measured from the intertragal notch) to ensure proximity to the tympanic membrane. The system was calibrated before each session, and a loudspeaker was positioned at 45° azimuth at a distance of 30 cm.

Real ear aided responses were recorded using a 65 dB SPL International Speech Test Signal (ISTS). Measurements were taken after the output curve stabilized. Frequency specific outputs were then converted to A-weighted levels by applying standard A-weighting correction values because regulatory exposure limits are expressed in dBA [19]. The A-weighted values across frequency bands were logarithmically summed to obtain a single overall A-weighted sound pressure level (dBA) for each participant. This overall dBA value served as an estimate of the intensity of hearing-aid output delivered to the ear canal under standardized conditions.

Finally, an 8-h equivalent continuous exposure level (*Leq_8h_*) was calculated using Equation (1) to estimate the potential daily noise dose associated with hearing aid use and to permit comparison with occupational noise exposure guidelines [19].

Leq_8h_ = L_T_ + 10log_10_(T/8), (1)

where $L_{T}\;$ is the overall A-weighted real-ear level measured in the laboratory condition, and T is the participant-reported average daily duration of hearing-aid use (in hours). This transformation enabled the approximation of cumulative daily exposure and permitted comparison with established occupational damage-risk criteria, similar to approaches used in studies examining exposure from personal listening devices [19,20,21].

Later, during the analysis the hearing aid users were sub-grouped using a classification similar to the damage risk criteria (DRC) based method used by Sing and Sasidharan [19]. Users with Leq8h ≥ 85 dBA were categorized as the ‘above DRC hearing aid users group (A-HA)’, and those with Leq8h < 85 dBA formed the ‘below DRC hearing aid users group (B-HA)’. Non-users (NU) constituted the third comparison group.

### 2.3. Recording of cVEMP, oVEMP, and vHIT

cVEMP, oVEMP, and vHIT are widely used non-invasive tools to assess the vestibular function. These three tests assess the saccule, utricle, and semicircular canals respectively [22,23].

The cVEMP and oVEMP were recorded from all participants using IHS SmartEP evoked potential system (Intelligent Hearing Systems, Miami, FL, USA). The cVEMP was used to assess the integrity of saccule and sacculocollic pathway and the oVEMP was used to assess the utricle and utriculo-ocular pathway. Both the cVEMP and oVEMP were elicited using 500 Hz tone burst with rarefaction polarity, which were presented at a repetition rate of 5.1/s. The stimuli were delivered to the test ear of participants at 125 dB peSPL using ER-3C insert earphones. Participants were seated in an upright and relaxed position during the recording of VEMP. The electrode sites were cleaned using skin preparation gel, and gold-plated electrodes were attached to the recording site using conduction paste. The absolute impedance of all electrodes was less than 5 kΩ and inter-electrode impedance was less than 2 kΩ. A total of 200 sweeps were averaged in each recording, in a response analysis window of 60 ms. Each recording was replicated twice to ensure reproducibility and reliability of the VEMP responses.

For recording the cVEMP, the non-inverting electrode was placed on the upper third of the ipsilateral sternocleidomastoid (SCM) muscle and the inverting electrode at the sternoclavicular junction. The ground electrode was placed on the forehead. While acquiring the response, participants turned the head to the opposite direction (contralateral side) of the test ear, to activate the ipsilateral SCM muscle. The electromyographic activity of the SCM muscle was continuously monitored between 50 and 150 µV for all the subjects using the EMG level feedback system available in the IHS SmartEP system during VEMP acquisition to ensure appropriate muscle activation and response quality. The response filter was set to 30–1500 Hz and the amplification gain was set at 5000.

To record the oVEMP, the non-inverting electrode was placed 1–2 cm below the contralateral eye, the inverting electrode was placed on the cheek directly below the non-inverting electrode, and the ground electrode was placed on the forehead. Participants were instructed to maintain an upward gaze at a point approximately 30 degrees above the eye level, fixed on the wall of the test room directly in front of the participants’ seat. The EMG level was set between 1 and 50 µV in the system for all the subjects. The response filter was set to 1–1000 Hz, and the amplification gain to 50,000. The remaining stimulus and acquisition parameters for oVEMP were similar to the cVEMP.

The peaks of VEMP waveforms were visually identified and marked by an experienced audiologist. For the cVEMP, absolute latencies of P1 and N1 peaks and the peak-to-peak amplitude of the P1–N1 complex were extracted. For oVEMP, the absolute latencies of the n1 and p1 peaks and the peak-to-peak amplitude of the n1–p1 complex were obtained.

vHIT was performed in a well-lit, quiet room using the EyeSeeCam™ vHIT system (Interacoustics A/S, Middelfart, Denmark). Participants were seated comfortably 1.5 m from a visual target placed at eye level on a light-colored wall. The vHIT goggles, equipped with motion sensors and a high-speed camera (mounted on the left side), were securely fitted to minimize slippage, and the lens was adjusted for optimal eye focus.

Eye and head calibrations were performed prior to testing. During eye calibration, a built-in laser array projected five dots (center, up, down, left, right) on the wall, and participants shifted their gaze as instructed. The vHIT software (version 3.1.0.205) verified calibration accuracy with an on-screen tick mark. Head calibration was then completed by moving the participant’s head horizontally and vertically as guided by real-time feedback; if calibration was inadequate, default settings were applied.

Participants were next asked to fixate on the target for 30 s to check for spontaneous nystagmus. Head impulses were delivered in three planes: lateral, LARP (Left Anterior-Right Posterior), and RALP (Right Anterior-Left Posterior). In the lateral plane, small positional amplitude (<15°) and high-velocity (150–300°/s) impulses were applied. For vertical canals, impulses were delivered at 100–300°/s. For each semicircular canal, 20 valid impulses were recorded. The software automatically rejected artifacts and impulses outside optimal velocity ranges, and a 3D animation provided real-time feedback to ensure accurate canal stimulation. The primary outcome measure was the vestibulo-ocular reflex (VOR) gain, calculated as the ratio of eye to head velocity from the regression function.

### 2.4. Data Analysis

Statistical analyses were performed using Jamovi software (version 2.3). Descriptive statistics were calculated for demographic variables and all vestibular outcome measures. The Shapiro–Wilk test and Levene’s test were used to assess normality and homogeneity of variances, respectively. As per their results, group differences in demographic variables such as age, duration of hearing loss, and pure-tone averages were examined using the Mann–Whitney U test. Comparisons of cVEMP parameters between hearing aid users and non-users were also performed using the Mann–Whitney U test. For oVEMP and vHIT comparison, an independent samples *t*-test (with Welch’s correction, where appropriate) was used.

Additional analysis between the above DRC hearing aid users (A-HA), below DRC hearing aid users (B-HA), and non-users (NU) groups was also performed. Because these data were not normally distributed, VEMP and vHIT parameters were compared using the Kruskal–Wallis test. Finally, multiple linear regression analyses were performed to determine whether duration of hearing aid use predicted vestibular outcomes after controlling for age, duration of hearing loss, hearing thresholds, gender, and other covariates.

## 3. Results

A total of 67 participants were included in the study and categorized into hearing aid users (*n* = 31) and non-users (*n* = 36). The hearing aid users group consisted of 20 males and 11 females, whereas the non-users group included 18 males and 18 females. As shown in Table 1, no significant group differences were observed for age or duration of hearing loss. Duration of hearing aid use was applicable only to the hearing aid users group, with a median of 4 years. Pure-tone averages also did not differ significantly between the groups. All participants demonstrated normal findings on subjective vestibular assessments. Oculomotor test results were consistent with the clinic’s age-appropriate normative data. Together with detailed vestibular case histories, these results confirmed that none of the participants presented with any evident vestibular pathology or ongoing acute vestibular symptoms.

Analysis of the real ear measurement-based exposure estimation revealed 18 hearing aid users had Leq8h above DRC (A-HA), with median 89.4 dBA (Q1–Q3: 87.6–92.8). Rest 13 hearing aid users had Leq8h below DRC (B-HA group), with a median of 74.28 dBA (Q1–Q3: 68.20–81.21).

Descriptive statistics of cVEMP parameters are presented in Table 2 along with statistical test results. As the Shapiro–Wilk test indicated non-normal distribution, group comparisons were made using the Mann–Whitney U test. No significant differences were found for right-ear P1 latency, right-ear N1 latency, left-ear N1 latency, right-ear P1–N1 amplitude, or left-ear P1–N1 amplitude. A significant difference was observed only for left-ear P1 latency, with hearing aid users showing longer latencies than non-users (U = 78.5, *p* = 0.049).

Descriptive data for oVEMP parameters are summarized in Table 3. Independent Samples *t*-tests with Welch’s correction indicated no significant differences between groups for N1 latencies of the right and left ears, P1 latencies of the right and left ears, or N1–P1 amplitudes. Thus, none of the oVEMP measures showed significant group differences.

Table 4 provides descriptive statistics for vHIT VOR gain. Independent Samples *t*-tests showed no significant differences in VOR gain for any semicircular canal, indicating comparable VOR gain across hearing aid users and non-users.

Comparisons among NU, A-HA, and B-HA groups using the Kruskal–Wallis test revealed no statistically significant effects for any of the VEMP and vHIT measures examined. Finally, multiple linear regression analyses were performed to determine whether duration of hearing aid use predicted vestibular outcomes after controlling for age, duration of hearing loss, hearing thresholds, gender, and other covariates. Across all models, duration of hearing aid use did not emerge as a significant predictor of any vestibular measure.

## 4. Discussion

Several studies have linked the increased risk of falls to hearing loss among individuals with SNHL. Following the publication of reviews and original studies linking increased risk of falls among individuals with sensorineural hearing loss [24]. Further, a substantial body of literature has noted a positive role of hearing aids in enhancing balance and postural stability [17,25,26,27]. In contrast to this prevailing area of focus, the present study explored a relatively underexamined area of the relationship between hearing aid use and vestibular function. As sound amplifiers, hearing aids deliver high-level auditory input to the ear. Previous research has established that prolonged or repeated exposure to high-intensity sounds, whether from occupational noise or personal listening devices, can result in vestibular damage [19,20,21,28]. Moreover, a recent preliminary study found variations in objective vestibular findings due to vibroacoustic stimulation from bone conduction hearing devices [29]. The present study was motivated by the paucity of empirical research evidence on the potential adverse vestibular effects associated with conventional hearing aid use, and, to the best of our knowledge, it is among the first to explore this possibility.

Findings from users of personal listening devices and animal models, which demonstrate measurable vestibular alterations and morphological changes in otolithic organs even after exposure to relatively low-intensity sound, provide a rationale for hypothesizing that exposure to amplified sound from high-power hearing aids may similarly induce subtle vestibular changes over time. However, the present findings do not support this hypothesis. For cVEMP, all parameters except for the left-ear P1 latency were statistically comparable between hearing aid users and non-users. Although the prolonged left-ear P1 latency in hearing aid users reached statistical significance, the magnitude of difference was small, remained within clinically accepted normal limits, and was not mirrored in the right ear or in any other cVEMP parameter. Moreover, oVEMP and vHIT findings showed no significant group differences in any of the parameters, suggesting intact utriculo-ocular pathway and semicircular canal function in long-term hearing aid users.

To examine whether exposure levels may play a role, hearing aid users were categorized into low-output and high-output groups based on real ear measurements converted to 8-h equivalent noise exposure levels. Even with this stratification, no significant differences emerged across the three groups in any cVEMP, oVEMP, or vHIT parameter. These results suggest that variations in hearing aid output within the range typically encountered in clinical practice are unlikely to contribute to measurable vestibular differences.

Regression analyses also indicated that duration of hearing aid use was not a significant predictor of vestibular outcomes when controlling for age, degree and duration of hearing loss, and other covariates. This further reinforces the notion that long-term use of appropriately fitted hearing aids does not adversely affect vestibular function. Notably, these findings aligned with studies reporting improved balance performance with hearing aid use, although those studies generally assessed short-term effects attributed to enhanced auditory cues rather than long-term vestibular physiology.

Several factors may help explain the absence of detrimental vestibular effects in this study. Modern hearing aids incorporate output-limiting algorithms, frequency-specific gain shaping, and advanced compression systems that reduce the likelihood of sustained high-level stimulation [9]. Although hearing aid outputs can reach high peak levels, real-world exposure typically fluctuates and remains well below the thresholds associated with vestibular injury in experimental models. It is also worth noting here that all participants in this study used hearing aids that were fitted in well-equipped audiology clinics by qualified audiologists, reducing the likelihood of output levels exceeding safe limits. Additionally, it is possible that central vestibular compensation may obscure peripheral vestibular damage as reflected in vestibular function tests. Central vestibular compensation is a well-recognized adaptive process that occurs following peripheral vestibular damage and reflects changes in vestibular symptoms and vestibular function tests [30,31,32]. If vestibular damage were to occur as a consequence of prolonged hearing aid use, it would likely be slow and progressive, a pattern that may allow sufficient time for effective central compensatory mechanisms to develop. As a result, overt vestibular symptoms such as dizziness or marked abnormalities on vestibular function tests may not be evident. This hypothesis remains speculative and highlights the need for further studies to provide empirical evidence addressing the potential role of central compensation in this context. The predominant presbycusis and idiopathic etiologies represented in this sample may also inherently limit the likelihood of vestibular involvement beyond age-related or idiopathic effects.

From a clinical perspective, the present study addresses an important question frequently encountered by audiologists during hearing aid counseling: whether the use of hearing aids could adversely affect the preserved structures of the inner ear. While evidence exists to suggest that appropriately fitted hearing aids do not compromise residual auditory function [11,33], empirical data addressing the findings of the present study suggest that, under controlled conditions, appropriately fitted hearing aids are unlikely to cause damage to peripheral vestibular organs. Their potential impact on the vestibular system has been limited.

This study also underscores the importance of professional hearing aid fitting, real-ear verification, and appropriate output management in clinical practice. However, audiologists should remain vigilant in monitoring vestibular symptoms, particularly in older adults or individuals with comorbid conditions, and should consider referral for vestibular assessment if symptoms such as dizziness or imbalance are reported. Overall, the findings support the continued clinical use of hearing aids as a safe intervention from a vestibular standpoint, while highlighting the need for individualized follow-up and future longitudinal research.

Nonetheless, certain limitations merit consideration. First, the cross-sectional design of the present study inherently limits the ability to establish causal relationships. It precludes temporal sequencing between hearing aid use and vestibular outcomes, as observations were made at a single time point. Moreover, this design is vulnerable to unmeasured or residual confounding. Factors such as intersubject variability in vestibular function, particularly VEMP amplitude, differences in the duration and patterns of hearing aid use, comorbid conditions, subclinical vestibular dysfunction, and variability in central vestibular mechanisms could not be fully controlled and may have influenced the observed findings. An ideal study design would therefore be longitudinal, with hearing aid users assessed prior to fitting and followed at multiple time points after hearing aid use. Inclusion of a matched control group would further help to control confounding factors, including age-related effects.

The second limitation relates to the relatively small sample size. Although it may be adequate for a preliminary evaluation, it may have limited statistical power and sensitivity to detect very subtle effects, particularly those with small effect sizes. Consequently, subtle vestibular alterations may not have been detected, especially in the presence of inter-individual variability and potential compensatory mechanisms.

Finally, although real ear aided measurements provide an accurate estimate of the sound pressure level delivered to the ear canal, the present method captures hearing aid output only for a single input level in a controlled laboratory setting. In everyday listening situations, hearing aid output varies dynamically depending on input level, acoustic environment, background noise, and the operation of adaptive processing features such as compression and noise management. Therefore, the REAR-based measurements obtained in this study may not fully represent the range of exposure that occurs during real-world hearing aid use. Quantifying real-life long-term exposure would require continuous logging of input levels or data logging of real ear output over extended periods, which was not feasible in the present study. As a result, a standardized laboratory-based protocol, similar to those used in personal listening devices exposure studies, was adopted as a practical and controlled approach for estimating potential exposure levels.

The present study included participants with moderately severe to profound degrees of hearing loss. Due to the limited sample size within each degree category, subgroup comparisons based on the severity of hearing loss were not performed. Future research is recommended to focus on narrower and more homogeneous categories, such as including only individuals with profound hearing loss. Because hearing aid output levels primarily depend on the degree of hearing loss, individuals with greater severity typically receive higher amplification. Consequently, long-term exposure to higher hearing aid output levels may pose a potential risk for vestibular dysfunction. Investigating this relationship within a more uniform hearing-loss category would therefore be a valuable direction for future studies.

Future research should employ longitudinal designs with larger sample sizes to enhance statistical power and improve sensitivity for detecting subtle or early vestibular changes that may not be evident in cross-sectional analyses. Prospective studies assessing vestibular function prior to hearing aid fitting and at multiple follow-up intervals would allow clearer evaluation of temporal relationships and potential cumulative effects of amplified sound exposure.

Additionally, future investigations should consider stratifying participants based on the severity of hearing loss, focusing on more homogeneous groups such as individuals with profound hearing loss who typically receive higher levels of amplification. Such refinement would improve generalizability and enable more precise examination of dose–response relationships between hearing aid output and vestibular function. Inclusion of matched control groups and real-world exposure monitoring through data logging or continuous input-level tracking would further strengthen the ecological validity of future studies. Finally, evaluating vestibular outcomes in individuals using hearing aids fitted without standardized audiological procedures may help delineate the role of fitting quality and output regulation in vestibular safety.

## 5. Conclusions

The present study, to the best of our knowledge, is among the first to examine the possibility of vestibular damage associated with the long-term use of high-power hearing aids. The rationale for this research question was mainly informed by established evidence on the effects of personal music devices on vestibular function. Contrary to these prior observations, the current study did not identify any significant differences in vestibular function between long-term hearing aid users and non-users, as assessed using cVEMP, oVEMP, and vHIT measures. Furthermore, neither hearing aid output levels nor duration of use were found to be associated with vestibular outcomes.

The absence of significant group differences should be interpreted with caution. Several factors discussed in the limitations section, including methodological constraints like cross-sectional study design, sample size, and potential confounding variables, may have obscured subtle or early vestibular changes related to long-term hearing aid use. Given the mixed evidence in the existing literature and the limitations of the present study, further research employing larger sample sizes, longitudinal designs, and more sensitive vestibular assessment tools is warranted to better understand the relationship between hearing aid use and vestibular function.

## Figures and Tables

**Table 1:** Comparison of participant characteristics between hearing aid users and non-users.

Characteristics	Hearing Aid Users	Non-Users	Mann–Whitney U
	Median (Q1–Q3)	Median (Q1–Q3)	*p* Value
Age (years)	56 (34.5–61.5)	54 (46.5–61.3)	*U* = 530.5, *p* = 0.734
Duration of Hearing Loss (years)	6 (4–8)	5 (3.75–8)	*U* = 442.0, *p* = 0.143
Duration of Hearing Aid Use (years)	4 (3–6)	Not applicable	Not applicable
Pure tone average-Right ear (dB HL)	73.8 (58.8–82.5)	69.4 (59.7–80)	*U* = 505.5, *p* = 0.513
Pure tone average-Left Ear (dB HL)	75 (63.1–81.3)	66.3 (58.4–80)	*U* = 497.0, *p* = 0.446

**Table 2:** Comparison of saccular function based on cVEMP peak latencies and peak-to-peak amplitudes between hearing aid users and non-users.

Parameter	Ear	Hearing Aid Users		Non-Users		Mann–Whitney U, *p*-Value
		Mean (SD)	Median (Q1–Q3)	Mean (SD)	Median (Q1–Q3)	
P1 Latency (ms)	Right	15.10 (1.72)	14.80 (13.60–16.10)	14.50 (1.62)	14.20 (13.60–15.80)	*U* = 138.0, *p* = 0.336
	Left	16.30 (2.49)	15.80 (14.90–16.80)	14.90 (1.57)	14.40 (14.00–15.50)	*U* = 78.5, *p* = 0.049 *
N1 Latency (ms)	Right	22.00 (4.31)	22.10 (21.00–23.90)	21.90 (1.96)	22.40 (20.80–23.60)	*U* = 146.0, *p* = 0.473
	Left	21.80 (2.84)	21.80 (20.70–23.40)	22.30 (2.23)	21.60 (20.60–24.20)	*U* = 132.5, *p* = 1.000
P1-N1 Amplitude (μV)	Right	38.40 (19.60)	36.70 (22.50–53.10)	31.20 (16.80)	25.30 (20.80–41.30)	*U* = 146.0, *p* = 0.474
	Left	35.50 (20.90)	26.30 (21.50–51.50)	36.90 (20.20)	30.20 (20.60–50.50)	*U* = 131.0, *p* = 0.956

Note: cVEMP = cervical vestibular evoked myogenic potential; SD = standard deviation; * *p* < 0.05.

**Table 3:** Comparison of utricular function based on oVEMP peak latencies and peak-to-peak amplitudes between hearing aid users and non-users.

Parameter	Ear	Hearing Aid Users		Non-Users		Welch’s *t*-Test, *p*-Value
		Mean (SD)	Median (Q1–Q3)	Mean (SD)	Median (Q1–Q3)	
N1 Latency (ms)	Right	12.00 (1.07)	12.00 (11.40–12.40)	12.50 (1.31)	11.90 (11.5–13.60)	*t* = 1.247, *p* = 0.224
	Left	11.90 (0.65)	11.80 (11.40–12.60)	12.40 (1.08)	12.00 (11.80–13.20)	*t* = 1.561, *p* = 0.135
P1 Latency (ms)	Right	16.60 (1.25)	16.80 (15.80–17.40)	17.20 (1.59)	17.30 (16.40–17.90)	*t* = 1.331, *p* = 0.196
	Left	16.81 (1.06)	16.60 (16.20–17.00)	17.00 (1.41)	16.60 (16.20–18.00)	*t* = 0.393, *p* = 0.698
N1-P1 Amplitude (μV)	Right	4.97 (3.71)	5.20 (1.70–7.20)	3.59 (2.58)	2.54 (1.70–5.20)	*t* = −1.301, *p* = 0.202
	Left	5.37 (3.60)	5.87 (3.00–6.70)	3.30 (3.03)	1.81 (1.20–4.10)	*t* = −1.589, *p* = 0.125

Note: oVEMP = ocular vestibular evoked myogenic potential; SD = standard deviation.

**Table 4:** Comparison of semicircular canal function based on vHIT VOR gain between hearing aid users and non-users.

SCC	Ear	Hearing Aid Users		Non-Users		Independent-Samples *t*-Test, *p* Value
		Mean (SD)	Median (Q1–Q3)	Mean (SD)	Median (Q1–Q3)	
LSCC	Right	0.928 (0.228)	0.940 (0.778–1.11)	0.919 (0.225)	0.915 (0.800–1.03)	*t* = −0.170, *p* = 0.870
	Left	0.870 (0.201)	0.895 (0.712–1.00)	0.867 (0.223)	0.885 (0.762–1.00)	*t* = −0.061, *p* = 0.950
PSCC	Right	0.846 (0.236)	0.880 (0.745–0.965)	0.831 (0.182)	0.845 (0.750–0.940)	*t* = −0.282, *p* = 0.781
	Left	0.814 (0.149)	0.830 (0.715–0.925)	0.787 (0.208)	0.820 (0.635–0.935)	*t* = −0.592, *p* = 0.550
ASCC	Right	0.812 (0.144)	0.800 (0.710–0.895)	0.837 (0.119)	0.845 (0.750–0.892)	*t* = 0.790, *p* = 0.432
	Left	0.785 (0.219)	0.810 (0.700–0.925)	0.802 (0.185)	0.80 (0.680–0.950)	*t* = 0.340, *p* = 0.731

Note: vHIT = video head impulse test; VOR = vestibulo-ocular reflex; SCC = Semicircular canal; LSCC = Lateral Semicircular canal; PSCC = Posterior Semicircular canal; ASCC = Anterior Semicircular canal.

## Data Availability

The data that support the findings of this study are not openly available due to reasons of sensitivity and are available from the corresponding author upon reasonable request.

## Abbreviations

The following abbreviations are used in this manuscript:

VEMP	Vestibular Evoked Myogenic Potential
cVEMP	Cervical Vestibular Evoked Myogenic Potential
oVEMP	Ocular Vestibular Evoked Myogenic Potential
vHIT	Video head impulse test
VOR	Vestibulo-ocular reflex
SNHL	Sensorineural Hearing loss
VNG	Videonystagmography
DHI	Dizziness Handicap Inventory
SCM	Sternocleidomastoid
SCC	Semicircular canal
LSCC	Lateral Semicircular canal
PSCC	Posterior Semicircular canal
ASCC	Anterior Semicircular canal
LARP	Left Anterior Right Posterior
RALP	Right Anterior Left Posterior
